# Extra-Pulmonary Vein Triggers at *de novo* and the Repeat Atrial Fibrillation Catheter Ablation

**DOI:** 10.3389/fcvm.2021.759967

**Published:** 2021-11-04

**Authors:** Daehoon Kim, Taehyun Hwang, Min Kim, Hee Tae Yu, Tae-Hoon Kim, Jae-Sun Uhm, Boyoung Joung, Moon-Hyoung Lee, Hui-Nam Pak

**Affiliations:** Division of Cardiology, Department of Internal Medicine, Yonsei University Health System, Seoul, South Korea

**Keywords:** atrial fibrillation, catheter ablation, recurrence, extra-pulmonary vein triggers, remodeling

## Abstract

**Background:** Extra-pulmonary vein triggers can play a significant role in atrial fibrillation recurrence after catheter ablation. We explored the characteristics of the extra-pulmonary vein (PV) triggers in *de novo* and repeat atrial fibrillation (AF) catheter ablation (AFCA).

**Methods:** We included 2,118 patients who underwent a *de novo* AFCA (women 27.6%, 59.2 ± 10.9 years old, paroxysmal AF 65.9%) and 227 of them conducted repeat procedures. All included patients underwent isoproterenol provocation tests at the end of the procedure, and then we analyzed extra-PV triggers-related factors.

**Results:** Extra-PV triggers were documented in 11.7% of patients undergoing *de novo* AFCA (1.22 ± 0.46 foci per patient) and 28.6% undergoing repeat AFCA (1.49 ± 0.73 foci per patient). Older age and higher LA volume index in *de novo* procedures and women, diabetes, and higher parasympathetic nerve activity (heart rate variability) in repeat-AFCA were independently associated with the existence of extra-PV triggers. The septum (19.9%), coronary sinus (14.7%), and superior vena cava (11.2%) were common extra-PV foci. Among 46 patients who were newly found to have mappable extra-PV triggers upon repeat procedures, 15 (32.6%) matched with the previous focal or empirical extra-PV ablation sites. The rate of AF recurrence was significantly higher in patients with extra-PV triggers than in those without after *de novo* (HR 1.91, 95% CI 1.54–2.38, *p* < 0.001) and repeat procedures (HR 2.68, 95% CI 1.63–4.42, *p* < 0.001).

**Conclusions:** Extra-PV triggers were commonly found in AF patients with significant remodeling and previous empirical extra-PV ablation. The existence of extra-PV triggers was independently associated with poorer rhythm outcomes after the *de novo* and repeat AFCA.

## Introduction

Catheter ablation is an effective treatment for atrial fibrillation by reducing the number of acute episodes and prolongs the duration of sinus rhythm, thereby improving the quality of life (1). Circumferential pulmonary vein (PV) isolation is considered to be the cornerstone technique of atrial fibrillation (AF) catheter ablation (AFCA) (2). Reports of AF recurrence rates after initial ablation procedures have been variable, ranging from 20 to 80% in several studies, and 30–70% of patients require a repeat ablation procedure to achieve sinus rhythm during long-term follow-up (3, 4). Previous studies have reported that the mechanism of arrhythmia recurrence after AFCA has a PV origin due to PV reconnections (5, 6). However, in especially persistent AF (PeAF), non-PV triggers play important roles in the pathophysiology through the progression of atriomyopathy and circumferential PV isolation (CPVI) alone generally does not achieve a satisfactory clinical outcome (7–11). The study of Kim et al. found that a larger number of reconnected PVs were paradoxically associated with a lower rate of arrhythmia recurrence after the second AF ablation (12). However, it is unclear whether the existence of extra-PV triggers is directly associated with AF recurrence at the condition of well-maintained PV isolation or after extensive empirical extra-PV ablations. The relationships between extra-PV triggers and multiple known pre-disposing factors or a higher recurrence after *de novo* or redo ablations have not been investigated. It is also unknown whether extensive empirical extra-PV ablation increases extra-PV foci by increasing the atrial damage (13). Therefore, we conducted a comprehensive analysis on the extra-PV triggers in patients who underwent a consistent isoproterenol provocation protocol during AFCA procedures, and assessed their characteristics and the association with the outcomes after both *de novo* and repeat ablation procedures. The purpose of this study was to compare the characteristics and mechanisms of extra-PV triggers and their role in the long-term outcome of AFCA.

## Materials and Methods

### Study Population

**Figure 1:**
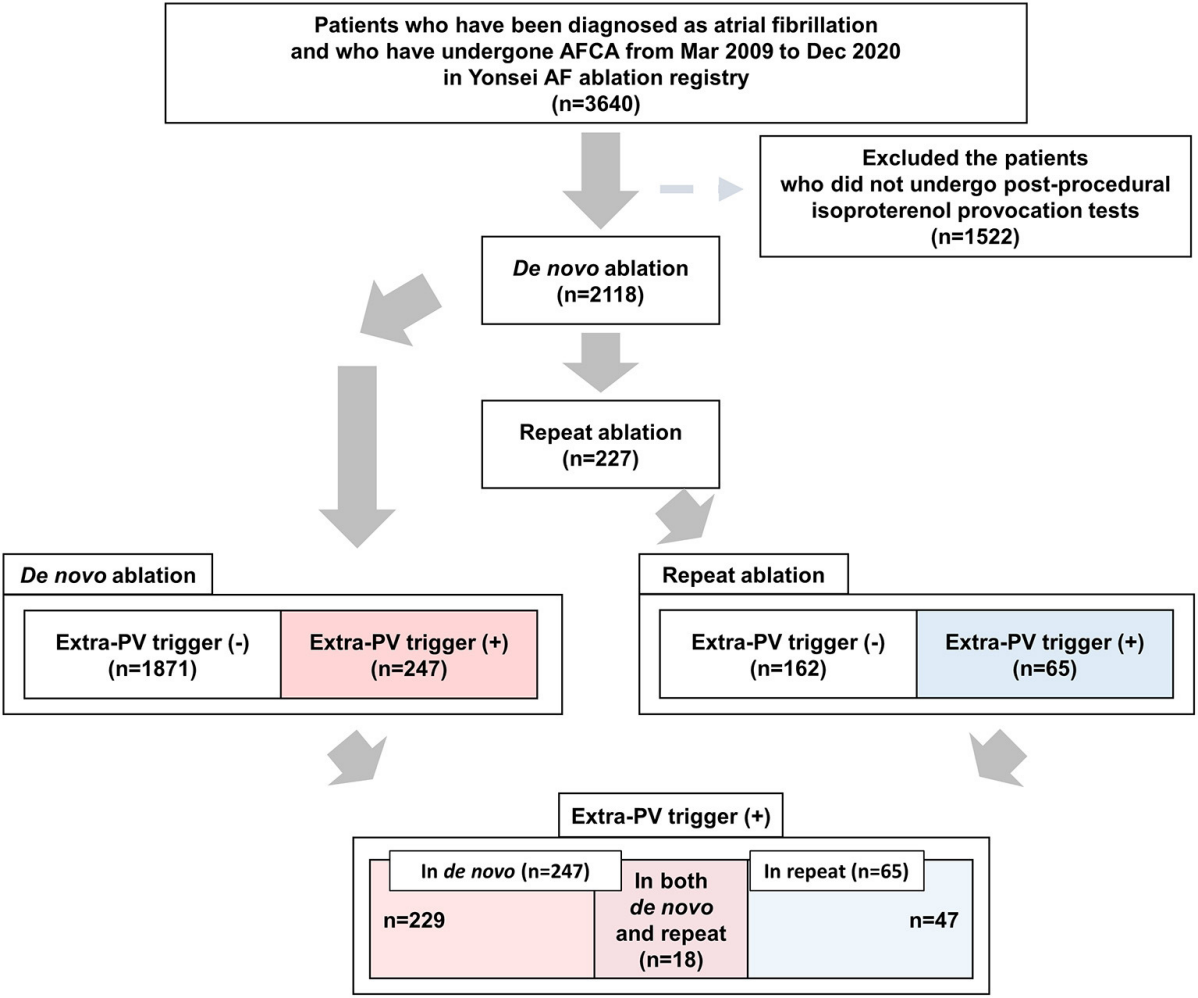
Flow chart of the present study. AFCA, atrial fibrillation catheter ablation; PV, pulmonary vein.

This study was performed as a single-center retrospective cohort study. The study protocol adhered to the principles of the Declaration of Helsinki and was approved by the Institutional Review Board at Yonsei University Health System. All patients provided written informed consent for inclusion in the Yonsei AF Ablation Cohort (ClinicalTrials.gov identifier: NCT02138695). Among 3,640 patients who underwent a *de novo* AFCA from March 2009 to December 2020, we included 2,118 patients who underwent post-procedural isoproterenol provocation tests. Among them, 227 patients underwent a repeat AFCA (Figure 1). The study exclusion criteria were as follows: (1) permanent AF refractory to electrical cardioversion; (2) AF with valvular disease grade > 2; (3) prior cardiac surgery with a concomitant AF surgery. Before the ablation procedure, the anatomies of the left atrium (LA) and PVs were visually defined on three-dimensional (3D) computed tomography (CT) (64 Channel, Light Speed Volume CT; Brilliance 63; Philips, Best, The Netherlands). We confirmed the absence of any LA thrombi by transesophageal echocardiography, intracardiac echocardiography, or CT. All antiarrhythmic drugs (AADs) were discontinued for at least five half-lives, and amiodarone was stopped at least 4 weeks before the procedure.

### Echocardiographic Evaluation

All included patients underwent transthoracic echocardiography (Sonos 5500, Philips Medical System, Andover, MA, USA or Vivid 7, GE Vingmed Ultrasound, Horten, Norway) before undergoing AFCA and at 1 year after the procedure. We acquired the cardiac chamber size, left ventricular ejection fraction, trans-mitral Doppler flow velocity, the ratio of the early diastolic peak mitral inflow velocity, and early diastolic mitral annular velocity (E/Em), following the American Society of Echocardiography guidelines.

### Electrophysiological Mapping and AF Catheter Ablation

We recorded the intracardiac electrograms using the Prucka CardioLab™ Electrophysiology system (General Electric Medical Systems, Inc., Milwaukee, WI, USA) and generated 3D electroanatomical maps (NavX, Abbott, Inc., Chicago, IL, USA; CARTO system, Biosense Webster, Diamond Bar, CA, USA) using a circumferential PV-mapping catheter (AFocus, Abbott, Inc., Chicago, IL, USA; Lasso, Biosense-Webster Inc., Diamond Bar, CA, USA) through a long sheath. The 3D geometries of the LA and PVs were generated using the 3D mapping system and then merged with 3D spiral CT images. Blinded to the patient information, a technician analyzed the color-coded CT-merged voltage maps. Then, we performed transseptal punctures. The number of transseptal punctures (single vs. double) was at the discretion of the operator. Afterward, we obtained multi-view pulmonary venograms for the perfect matching of 3D-map, CT, and fluoroscopy in all patients except for significant renal disease. Immediately after the transseptal puncture, systemic anticoagulation was started with an intravenous bolus of heparin 200 IU/kg followed by intermittent boluses to maintain an activated clotting time of 350–400 s.

The details of the AFCA technique and strategy were described previously (12). An open-irrigated tip catheter (Celsius, Johnson & Johnson Inc., Diamond Bar, CA, USA; NaviStar ThermoCool, Biosense Webster Inc., Diamond Bar, CA, USA; ThermoCool SF, Biosense Webster Inc.; ThermoCool SmartTouch, Biosense Webster Inc.; Coolflex, Abbott Inc., Minnetonka, MN, USA; 30–35 W; 47°C; FlexAbility, Abbott Inc.; ThermoCool SmartTouch, Biosense Webster Inc., and TactiCath, Abbott Inc.) was used for the AFCA. Because we included patients over a relatively long period for this study, the radiofrequency power for the AFCA varied between 25 and 60 W. The endpoint of ablation at each site was as the average impedance drop >10% of baseline or an >80% decrease in the local electrogram voltage amplitude. We generated a CPVI with a bidirectional block in all patients. Most patients (91.6%) underwent the creation of cavotricuspid isthmus block during the *de novo* procedure unless there was atrioventricular conduction disease. Empirical linear ablation, including a roofline, posterior inferior line (posterior box lesion), and anterior line, left lateral isthmus ablation, right atrial ablation, or complex fractionated electrogram ablation were performed at the discretion of the operator.

### Isoproterenol Provocation and Ablation Endpoint

After completing the protocol-based ablation, AF or atrial tachycardia (AT) was induced by 10-s high-current burst pacing (10 mA, pulse width 5 ms, Bloom Associates, Denver, CO, USA) from the high right atrial (RA) electrodes. This commenced at a pacing cycle length of 250 ms and was gradually reduced to 120 ms as previously described in the procedure (14). We infused isoproterenol (5–20 μg/min depending on ß-blocker use with a target heart rate of 120 bpm) for at least 3 min before induction and maintained this for 3 min after the induction of AF or AT. If sustained AF or AT was induced, internal cardioversion was performed by utilizing biphasic shock (2–20 J) with R wave synchronization (Lifepak12, Physiocontrol Ltd., Redmond, WA, USA). We conducted all the procedures under conscious sedation but induced deep sedation immediately before electrical cardioversion. We ended the procedure when there was no immediate recurrence of AF within the 10 min after the isoproterenol infusion with or without cardioversion. If further AF triggers were observed under the isoproterenol effect, we determined the potential location of the extra-PV triggers based on the contact bipolar electrograms and conducted a quick and detailed 3D-activation mapping with a multielectrode catheter. Based on the 3D mapping of the non-PV foci, we ablated those foci with 35–50 W for 10 s in each lesion until elimination. After the first round ablation, we performed the provocation procedure if it was a highly reproducible extra-PV trigger. However, we did not conduct the second time isoproterenol provocation in general. We defined the extra-PV foci as AF triggering points by isoproterenol provocation after the bidirectional block of CPVI.

### Post-ablation Management and Follow-Up

We discharged the patients without taking any AADs except for those who had recurrent extra-PV triggers after the AFCA procedure, symptomatic frequent atrial pre-mature beats, non-sustained atrial tachycardia, or early recurrence of AF on telemetry during admission. Patients visited the outpatient clinic regularly at 1, 3, 6, and 12 months and then every 6 months or whenever symptoms occurred. All patients underwent ECG during every visit and 24-h Holter recordings at 3 and 6 months and every 6 months for 2 years, annually for 2–5 years, and then biannually after 5 years. Holter monitoring or event monitor recordings were obtained when patients reported palpitations suggestive of an arrhythmia recurrence. We defined an AF recurrence as any episode of AF or AT of at least 30 s in duration. Any ECG documentation of an AF recurrence within a 3-month blanking period was diagnosed as an early recurrence, and an AF recurrence occurring more than 3 months after the procedure was diagnosed as a clinical recurrence. We analyzed the clinical recurrences depending on the existence of extra-PV triggers and their locations and risk factors.

### Holter Monitor Records and Heart Rate Variability Analysis

A GE Marquette MARS 8000 Holter analyzer (General Electric Medical Systems, Chicago, IL, USA) was used to analyze heart rate variability (HRV) based on the 24-h Holter monitor recordings. Pre-mature ventricular beats, pre-mature atrial beats, and electrical artifacts were excluded from the analysis. The mean heart rate and the following time-domain HRV parameters were analyzed as follows: mean RR interval (mean NN interval), SD of NN intervals, SD of the 5 min mean of NN intervals, and root mean square of differences between successive NN intervals (rMSSD). The following parameters were calculated as follows: very-low-frequency components (<0.04 Hz), low-frequency components (LF; 0.04–0.15 Hz), high-frequency components (HF; 0.15–0.4 Hz), and LF:HF ratio. The HF and rMSSD were indicators of parasympathetic nervous activity.

### Statistical Analysis

Continuous variables were summarized as the *M* ± *SD* and compared by independent two-sample *t*-test analysis. Categorical variables were summarized as the number (percentage of the group total) and compared by either the chi-square test or Fisher's exact-test. Multivariable logistic regression was applied to identify predictors associated with the existence of extra-PV triggers. Kaplan–Meier analysis with the log-rank-test was used to calculate AF recurrence-free survival over time and to compare recurrence rates according to the existence of extra-PV triggers. A multivariable Cox regression analysis was performed to identify the predictors associated with a clinical recurrence of AF. A two-sided *P* < 0.05 was considered statistically significant. The statistical analyses were performed using R version 4.0.2 software (The R Foundation, www.R-project.org, Vienna, Austria).

## Results

### Characteristics of Extra-PV Triggers

Table 1 summarizes the baseline clinical characteristics of the patients with extra-PV triggers during the *de novo* and repeats AFCA procedures. Regarding *de novo* procedures, the patients with an extra-PV trigger tended to be women and older and to have a longer AF duration, lesser history of vascular disease, higher LA volume indices on the echocardiogram, lower mean LA voltage, and lower pericardial fat volume. Details of procedural complications are presented in Supplementary Tables 1, 2. In the multivariable logistic regression, an older age [odds ratio (OR) 1.02 per 1-year increase, 95% CI 1–1.03, *p* = 0.021], no history of vascular disease (OR 0.53, 95% CI 0.3–0.89, *p* = 0.023), and a higher LA volume index (OR 1.01, 95% CI 1–1.02, *p* = 0.015) were independently associated with the existence of extra-PV triggers during the *de novo* procedure (Table 2). Among the 247 patients who demonstrated an extra-PV trigger in the *de novo* procedure, 19.8% (49/247) underwent a repeat ablation.

**Table 1:** Baseline clinical and procedural characteristics according to the existence of extra-PV triggers.

	***De novo*** **AF ablation**				**Repeat AF ablation**			
	**Overall**	**Extra-PV triggers (–)**	**Extra-PV triggers (+)**	***P-*value**	**Overall**	**Extra-PV triggers (–)**	**Extra-PV triggers (+)**	***P-*value**
	**(*n* = 2,118)**	**(*n* = 1,871)**	**(*n* = 247)**		**(*n* = 227)**	**(*n* = 162)**	**(*n* = 65)**	
Age, years	59.1 ± 11.0	58.8 ± 11.0	60.9 ± 10.3	0.005	61.0 ± 9.9	60.7 ± 9.9	61.6 ± 10.1	0.522
Female, *n* (%)	584 (27.6)	495 (26.5)	89 (36.0)	0.002	63 (27.8)	37 (22.8)	26 (40.0)	0.014
Paroxysmal AF, *n* (%)	1,401 (66.1)	1,240 (66.3)	161 (65.1)	0.788	134 (59.0)	95 (58.6)	39 (60.0)	0.969
AF duration, months	38.1 ± 44.3	35.9 ± 42.3	51.4 ± 53.2	<0.001	75.9 ± 48.9	76.3 ± 48.6	74.9 ± 50.0	0.432
BMI, kg/m^2^	24.9 ± 3.0	24.9 ± 3.0	24.6 ± 2.9	0.112	25.0 ± 3.2	25.1 ± 3.2	24.6 ± 3.2	0.277
CHA_2_DS_2_-VASc score	1.77 ± 1.56	1.75 ± 1.55	1.91 ± 1.66	0.122	1.98 ± 1.52	1.88 ± 1.41	2.22 ± 1.75	0.137
**Comorbidities**, ***n*** **(%)**								
Heart failure	277 (13.1)	243 (13.0)	34 (13.8)	0.810	46 (20.3)	33 (20.4)	13 (20.0)	1.000
Hypertension	958 (45.2)	843 (45.1)	115 (46.6)	0.705	108 (47.6)	72 (44.4)	36 (55.4)	0.179
Diabetes mellitus	312 (14.7)	280 (15.0)	32 (13.0)	0.458	33 (14.5)	18 (11.1)	15 (23.1)	0.035
Stroke	245 (11.6)	214 (11.4)	31 (12.6)	0.683	33 (14.5)	26 (16.0)	7 (10.8)	0.417
Vascular disease	229 (10.8)	213 (11.4)	16 (6.5)	0.026	16 (7.0)	10 (6.2)	6 (9.2)	0.598
**Echocardiographic parameters**								
LA dimension, mm	41.3 ± 6.1	41.3 ± 6.1	41.2 ± 6.3	0.781	41.5 ± 5.9	41.9 ± 5.8	40.5 ± 6.0	0.103
LA volume index, ml/m^2^	37.4 ± 13.4	37.1 ± 13.4	40.0 ± 13.2	0.001	38.9 ± 13.2	38.9 ± 12.0	39.1 ± 15.8	0.913
LV ejection fraction, %	63.3 ± 8.2	63.2 ± 8.3	63.7 ± 7.3	0.345	62.7 ± 7.9	62.8 ± 7.6	62.5 ± 8.7	0.857
E/Em	10.2 ± 4.2	10.2 ± 4.2	10.5 ± 4.4	0.238	10.5 ± 4.2	10.4 ± 4.0	10.9 ± 4.7	0.408
LA volume by CT, ml	151.4 ± 45.7	150.7 ± 45.4	156.4 ± 48.2	0.072	167.9 ± 48.3	166.9 ± 44.0	170.2 ± 57.6	0.646
Pericardial fat volume, cm^3^	110.0 ± 56.1	111.4 ± 56.4	99.5 ± 53.3	0.008	116.8 ± 58.3	123.0 ± 60.4	102.5 ± 51.1	0.054
Mean LA voltage, mV (*n* = 1,594)	1.52 ± 0.71	1.54 ± 0.71	1.40 ± 0.70	0.015	1.22 ± 0.67	1.25 ± 0.68	1.14 ± 0.62	0.294
Procedure time, min	175.0 ± 53.6	174.2 ± 53.1	180.5 ± 57.4	0.086	137.6 ± 44.3	132.1 ± 41.7	151.4 ± 47.8	0.003
Ablation time, s	4,530 ± 1,878	4,545 ± 1,865	4,413 ± 1,979	0.297	1,987 ± 1,163	1,927 ± 1,145	2,138 ± 1,201	0.217
**Ablation lesions**, ***n*** **(%/BDB%)**								
CPVI	2,118 (100.0)	1,871 (100.0)	247 (100.0)	NA	227 (100.0)	162 (100.0)	65 (100.0)	NA
CTI ablation	1,940 (91.6)	1,724 (92.2)	216 (87.4)	0.016	212 (93.8)	151 (93.8)	61 (93.8)	1.000
Posterior box isolation	575 (27.2/60.9)	511 (27.3/61.1)	64 (26.0/59.4)	0.717	99 (43.8/51.5)	71 (44.1/53.5)	28 (43.1/46.4)	1.000
Anterior line	443 (20.9/63.9)	388 (20.7/67.0)	55 (22.3/85.9)	0.640	87 (38.3 /64.3)	62 (38.3/59.7)	25 (38.5/76.0)	1.000
Complications, *n* (%)	75 (3.5)	62 (3.3)	13 (5.3)	0.169	10 (4.4)	4 (2.5)	6 (9.2)	0.059
Major complications^*^, *n* (%)	34 (1.6)	28 (1.5)	6 (2.4)	0.408	5 (2.2)	2 (1.2)	3 (4.6)	0.285

* *Complications that resulted in permanent injury or death, required intervention, or a prolonged or required hospitalization for more than 48 h*.

*AAD, antiarrhythmic drug; AF, atrial fibrillation; AT, atrial tachycardia; BDB, Bidirectional block; BMI, body mass index; CPVI, Circumferential pulmonary vein isolation; CT, computed tomography; CTI, cavotricuspid isthmus; E/Em, mitral inflow velocity/mitral annulus tissue velocity; HF, high frequency; HRV, Heart rate variability; LA, left atrium; LF, low frequency; LV, left ventricle; NA, not applicable; PV, pulmonary vein; rMSSD, root mean square of successive differences between normal heartbeats*.

**Table 2:** Logistic regression analysis for predictors of extra-PV triggers in the *de novo* and repeat AFCA.

	**Extra-PV triggers in** ***de novo*** **ablation**				**Extra-PV triggers in repeat ablation**					
	**Univariable**		**Multivariable (Model 1)**		**Univariable**		**Multivariable (Model 1)**		**Multivariable (Model 2)**	
	**OR (95% CI)**	***P***	**OR (95% CI)**	***P***	**OR (95% CI)**	***P***	**OR (95% CI)**	***P***	**OR (95% CI)**	***P***
Age, yrs	1.02 (1.01–1.03)	0.005	1.02 (1.00–1.03)	0.021	1.01 (0.98–1.04)	0.520	1.00 (0.97–1.04)	0.768	1.02 (0.98–1.06)	0.309
Female	1.57 (1.18–2.07)	0.002	1.30 (0.96–1.74)	0.087	2.25 (1.21–4.18)	0.010	2.72 (1.39–5.38)	0.004	2.17 (1.03–4.58)	0.042
Paroxysmal atrial fibrillation	0.97 (0.74–1.29)	0.849	1.08 (0.80–1.48)	0.610	1.08 (0.60–1.97)	0.790	0.91 (0.47–1.77)	0.782	1.31 (0.62–2.85)	0.492
AF duration, month	1.01 (1.00–1.01)	<0.001			1.00 (0.99–1.01)	0.867				
BMI, kg/m^2^	0.96 (0.92–1.01)	0.112			0.95 (0.86–1.04)	0.276				
Congestive heart failure	1.07 (0.72–1.55)	0.733			0.98 (0.46–1.97)	0.950				
Hypertension	1.06 (0.81–1.39)	0.656			1.55 (0.87–2.78)	0.137				
Diabetes	0.85 (0.56–1.24)	0.403	0.81 (0.53–1.21)	0.323	2.40 (1.11–5.12)	0.023	2.42 (1.07–5.47)	0.033		
Stroke	1.05 (0.85–1.28)	0.607			0.79 (0.49–1.21)	0.311				
Vascular disease	0.54 (0.31–0.88)	0.021	0.53 (0.30–0.89)	0.023	1.55 (0.51–4.36)	0.419	1.69 (0.51–5.15)	0.366		
CHA_2_DS_2_-VASc score	1.07 (0.98–1.16)	0.123			1.15 (0.95–1.39)	0.138				
LA diameter, mm	1.00 (0.98–1.02)	0.780			0.96 (0.91–1.01)	0.104				
LAVI, ml/m^2^	1.01 (1.01–1.02)	0.001	1.01 (1.00–1.02)	0.015	1.00 (0.98–1.02)	0.912	0.99 (0.96–1.01)	0.259		
LVEF, %	1.01 (0.99–1.03)	0.345			1.00 (0.96–1.03)	0.856				
E/Em	1.02 (0.99–1.05)	0.238			1.03 (0.96–1.10)	0.408				
rMSSD after 3 months *de novo* AFCA					1.02 (1.01–1.04)	0.006			1.02 (1.00–1.04)	0.025
LF after 3 months *de novo* AFCA					1.02 (1.00–1.04)	0.071				
HF after 3 months *de novo* AFCA					1.05 (1.01–1.09)	0.009				
LF-HF ratio after 3 months *de novo* AFCA					1.60 (0.84–3.09)	0.151				
LA volume (by CT), ml	1.00 (1.00–1.01)	0.072			1.00 (1.00–1.01)	0.636				
Pericardial fat volume (by CT), ml	1.00 (0.99–1.00)	0.008			0.99 (0.99–1.00)	0.057				

*Variables used in the multivariable analyses: Model 1 includes age, sex, paroxysmal atrial fibrillation, diabetes, vascular disease, and LAVI; Model 2 includes age, sex, paroxysmal atrial fibrillation, and rMSSD*.

*AF, atrial fibrillation; CT, computed tomography; E/Em; ratio of the peak mitral flow velocity of the early rapid filling to the early diastolic velocity of the mitral annulus; HF, high frequency; LA, left atrium; LAVI, left atrial volume index; LF, low frequency; LVEF, left ventricular ejection fracture; rMSSD, root mean square of successive differences between normal heartbeats*.

The median interval between the *de novo* and repeat procedures was 2 (interquartile range 1.1–4.1) years. The patients with extra-PV triggers in repeat procedures were more likely to be women (*p* = 0.014) and have diabetes (*p* = 0.035, Table 1). Being a woman (OR 2.72, 95% CI 1.39–5.38, *p* = 0.004), having diabetes (OR 2.42, 95% CI 1.07–5.47, *p* = 0.033; Model 1), and having higher rMSSD (OR 1.02, 95 CI 1–1.04, *p* = 0.025; Model 2) were independently associated with the existence of extra-PV foci during the repeat procedure (Table 2).

### Locations of Extra-PV Triggers

**Figure 2:**
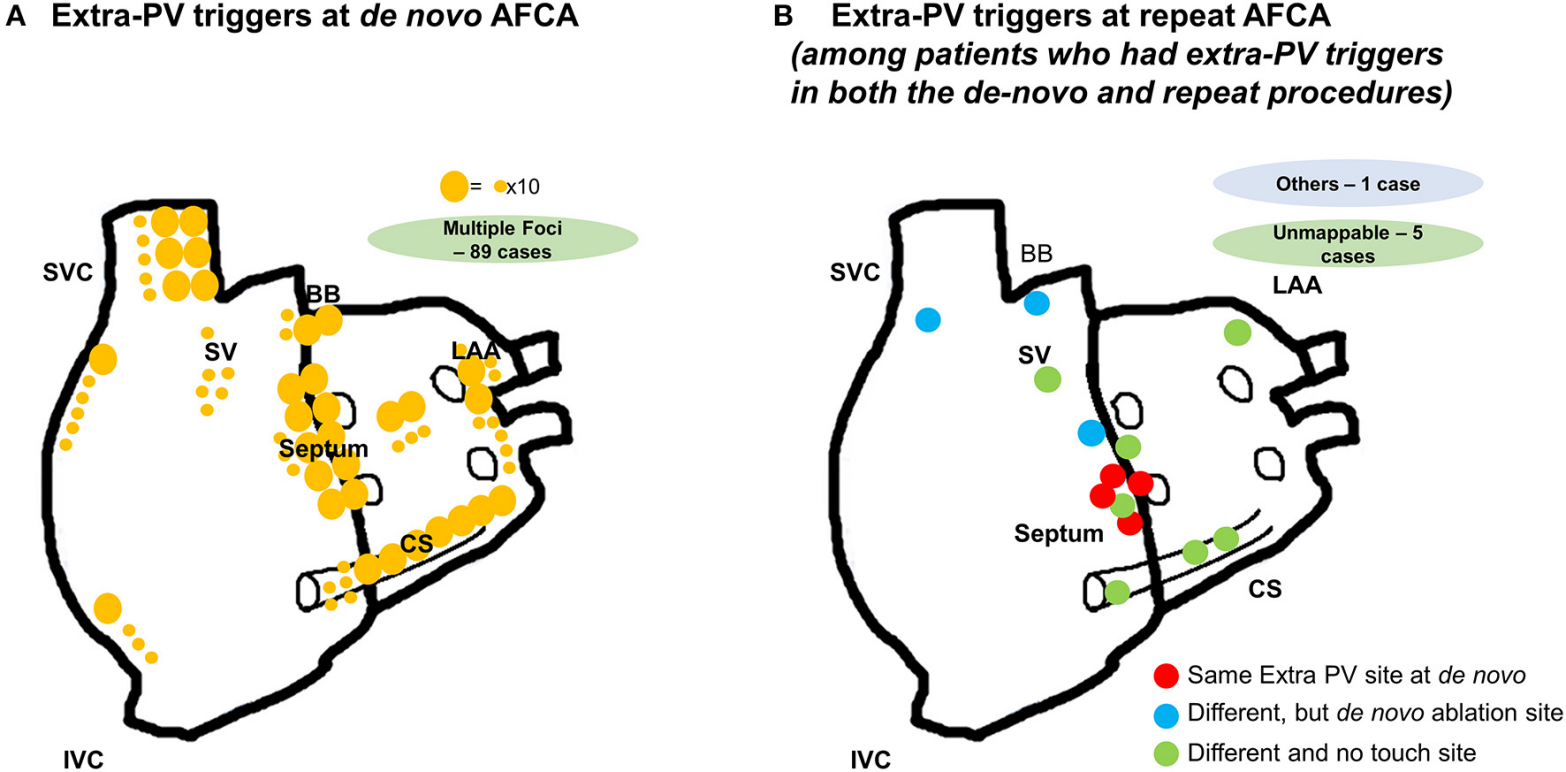
Locations of the extra-PV triggers **(A)** during the *de novo* AFCA; **(B)** during the repeat AFCA in patients who had both extra-PV triggers in the *de novo* and repeat AFCA.

The proportion of patients who had extra-PV triggers at the end of the procedure was 11.7% (247/2,118) during the *de novo* procedure and 28.6% (65/227) during the repeat procedure (*p* < 0.001). The number of extra-PV trigger foci was significantly higher in the repeat ablation (1.49 ± 0.73 per patient) than in *de novo* procedure (1.22 ± 0.46 per patient, *p* < 0.001; Supplementary Table 3). The location of the extra-PV triggers in the *de novo* and repeat ablation procedures are presented in Figure 2. The septum (19.9%), coronary sinus (14.7%), and superior vena cava (11.2%) were the most common sites for extra-PV triggers regardless of whether a *de novo* or repeat procedure (Supplementary Table 3). Multifocal extra-PV triggers were documented in 21.5% of patients and were more common in the repeat procedures (18.2% in *de novo* vs. 33.8% in repeat ablations, *p* = 0.006; Supplementary Table 3). Extra-PV triggers were unmappable in 6.5 and 9.2% of the patients in the *de novo* and repeat ablation procedures, respectively.

### Comparisons Between Extra-PV Triggers During the *de novo* vs. Repeat Procedures

Figure 2B displays the locations of the extra-PV foci in the repeat procedures in patients who showed in both *de novo* and repeat ablations. Table 3 summarizes the characteristics of the extra-PV triggers provoked in repeat procedures. Among the 65 patients revealed to have extra-PV triggers in redo procedures, 6.2% (4/65) had an origin at the same site as the extra-PV foci in the *de novo* procedure, while 84.6% (38/55) had new origins of extra-PV triggers. Among the 46 patients who were newly found to have mappable extra-PV triggers in the redo ablation, 15 (32.6%) patients had extra-PV trigger sites matched with the previous empirical extra-PV ablation sites, whereas 31 (67.4%) had extra-PV trigger sites which were not touched at *de novo* procedures.

**Table 3:** Characteristics of the extra-PV triggers provoked in repeat ablation procedures.

	**Extra-PV triggers in repeat procedures (*n* = 65)**	**Provoked only in the repeat procedures (*n* = 47)**
***De novo*** **lesion set**, ***n*** **(%)**		
CPVI alone, *n* (%)	28 (43.1%)	19 (40.4%)
Empirical LA ablation, *n* (%)	37 (56.9%)	28 (59.6%)
**Locations of extra-PV triggers at redo**		
Same extra-PV trigger in the *de novo* ablation, *n* (%)	4 (6.2%)	NA
New extra-PV trigger in the repeat ablation, *n* (%)	55 (84.6%)	46 (97.9%)
Sites not ablated at *de novo, n* (%)	38/55 (69.1%)	31/46 (67.4%)
Empirical extra-PV LA ablation sites at *de novo, n* (%)	17/55 (30.9%)	15/46 (32.6%)
Unmappable extra-PV triggers, *n* (%)	6 (9.2%)	1 (2.1%)

*CPVI, Circumferential pulmonary vein isolation; LA, left atrium; NA, not applicable; PV, pulmonary vein*.

### Extra-PV Triggers and the Rhythm Outcome After AFCA

**Figure 3:**
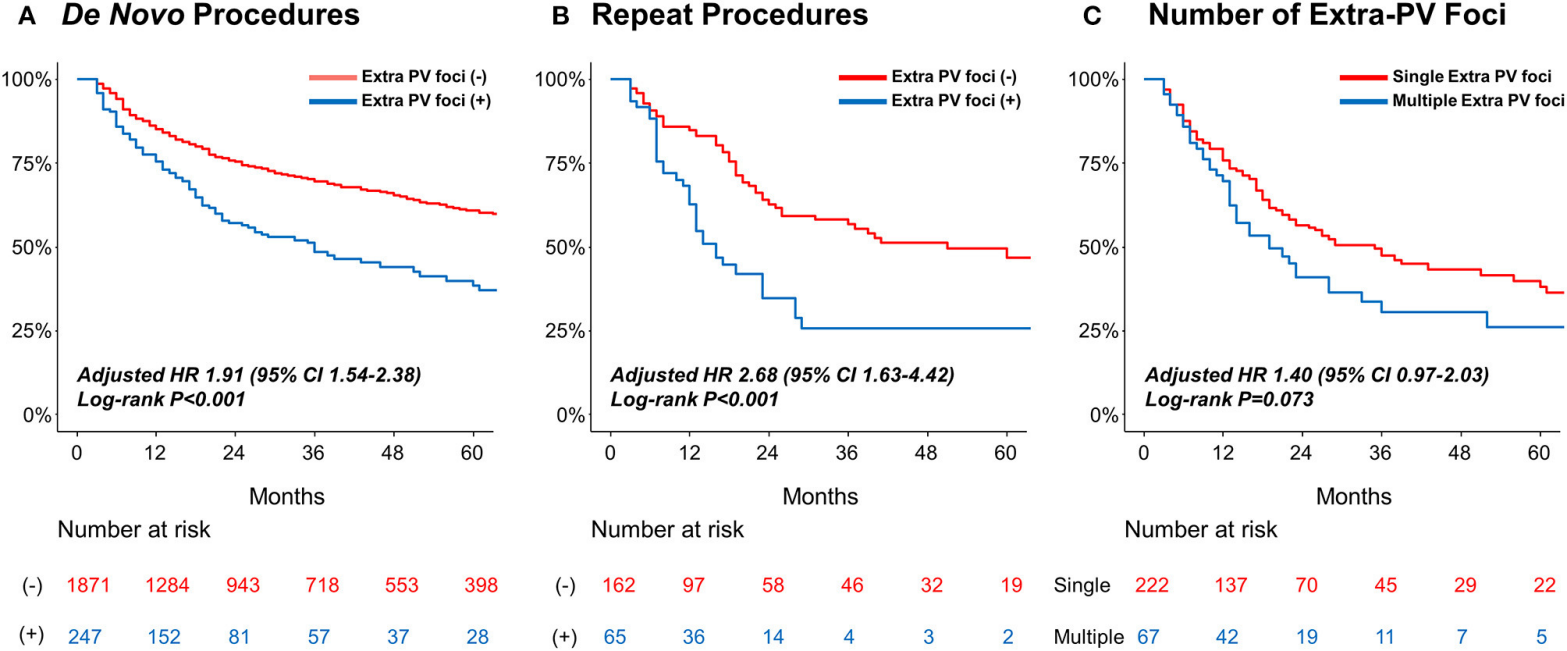
Kaplan–Meier analyses of the AF recurrence-free survival **(A)** according to extra-PV triggers in the *de novo* AFCA; **(B)** according to the extra-PV triggers in the repeat AFCA; and **(C)** according to the number of extra-PV triggers. AF, atrial fibrillation.

Table 4 summarizes the rhythm outcomes after *de novo* and repeat procedures according to the existence of extra-PV foci. AADs were maintained at discharge and 3 months after the repeat procedures more frequently in patients with extra-PV foci than in those without. During the mean follow-up of 50.2 ± 37.7 months, clinical recurrences of AF were significantly higher in the patients with extra-PV triggers (44.9%) than in those without (31.6%) after the *de novo* AFCA (Log-rank *p* < 0.001, Figure 3A). During a mean follow-up of 38.8 ± 29.5 months after the repeat ablation procedure, the rhythm outcome was consistently worse in patients with extra-PV triggers (57.5%) than in those without (36.7%) (Log-rank *p* < 0.001, Figure 3B). The comparison between the patients with a single extra-PV trigger and multiple extra-PV triggers is presented in Figure 3C (Log-rank *p* = 0.073). In the Cox regression analyses (Table 5), women [hazard ratio (HR) 1.33 95% CI 1.09–1.61, *p* = 0.004], persistent AF (HR 1.49, 95% CI 1.25–1.75, *p* < 0.001), increasing LA diameter (HR 1.04, 95% CI 1.02–1.06, *p* < 0.001), and the presence of extra-PV triggers (HR 1.91, 95% CI 1.54–2.38, *p* < 0.001) were independently associated with a clinical recurrence of AF after the *de novo* AFCA. The best cut-off value of LA diameter predicting recurrence was ≥44 mm (Supplementary Figure 1). The presence of extra-PV triggers (HR 2.68, 95% CI 1.63–4.42, *p* < 0.001) and persistent AF (HR 1.82, 95% CI 1.1–3.03, *p* = 0.019) were associated with an AF recurrence after the repeat procedure (Table 5).

**Table 4:** Clinical rhythm outcomes according to the existence of extra-PV triggers.

	***De novo*** **AF ablation**				**Repeat AF ablation**			
	**Overall**	**Extra-PV triggers (–)**	**Extra-PV triggers (+)**	***P-*value**	**Overall**	**Extra-PV triggers (–)**	**Extra-PV triggers (+)**	***P-*value**
	**(*n* = 2,118)**	**(*n* = 1,871)**	**(*n* = 247)**		**(*n* = 227)**	**(*n* = 162)**	**(*n* = 65)**	
Follow-up duration, months	50.2 ± 37.7	51.5 ± 37.9	40.5 ± 34.4	<0.001	37.3 ± 31.0	37.9 ± 31.5	35.8 ± 29.9	0.656
**AAD Use**, ***n*** **(%)**								
AADs at discharge	394 (18.6)	294 (15.7)	100 (40.5)	<0.001	65 (28.6)	39 (24.1)	26 (40.0)	0.025
AADs after 3 months	621 (31.2)	489 (28.0)	132 (54.1)	<0.001	94 (42.3)	57 (36.3)	37 (56.9)	0.007
Early recurrence, *n* (%)	565 (27.6)	447 (24.8)	118 (47.8)	<0.001	61 (26.9)	35 (21.6)	26 (40.0)	0.008
Clinical recurrence, *n* (%)	680 (33.2)	569 (31.6)	111 (44.9)	<0.001	94 (41.4)	57 (35.2)	37 (56.9)	0.004
At 12 months, *n* (%)	294 (14.4)	240 (13.3)	54 (21.9)	<0.001	43 (18.9)	22 (13.6)	21 (32.3)	0.002
At 24 months, *n* (%)	458 (22.4)	373 (20.7)	85 (34.4)	<0.001	78 (34.4)	44 (27.2)	34 (52.3)	0.001
AT Recurrence, *n* (% in recur/% in overall)	216 (31.8/10.2)	177 (31.1/9.5)	39 (35.1/15.8)	0.470	41 (43.6/18.1)	22 (38.6/13.6)	19 (51.4/29.2)	0.315
Cardioversion, *n* (% in recur/% in overall)	278 (40.9/13.6)	225 (39.5/12.5)	53 (47.7/21.5)	<0.001	46 (48.9/20.2)	30 (52.6/18.5)	16 (43.2/24.6)	0.498

*AAD, antiarrhythmic drug; AF, atrial fibrillation; AT, atrial tachycardia; PV, pulmonary vein*.

**Table 5:** Cox regression analysis of AF recurrence after the *de novo* and repeat ablation procedures.

	**Cox regression analysis of AF recurrence in the** ***de novo*** **ablation**				**Cox regression analysis of AF recurrence in the repeat ablation**			
	**Univariable**		**Multivariable**		**Univariable**		**Multivariable**	
	**HR (95% CI)**	***P***	**HR (95% CI)**	***P***	**HR (95% CI)**	***P***	**HR (95% CI)**	***P***
Age, yrs	1.01 (1.00–1.02)	0.027	1.01 (1.00–1.01)	0.314	1.00 (0.98–1.02)	0.738	0.97 (0.94–1.01)	0.104
Female	1.27 (1.09–1.50)	0.003	1.33 (1.09–1.61)	0.004	1.43 (0.92–2.21)	0.111	1.35 (0.77–2.38)	0.297
Persistent atrial fibrillation	1.79 (1.52–2.08)	<0.001	1.49 (1.25–1.75)	<0.001	1.72 (1.14–2.56)	0.009	1.82 (1.10–3.03)	0.019
AF duration, month	1.02 (1.00–1.00)	0.112			1.00 (1.00–1.01)	0.573		
BMI, kg/m^2^	1.02 (0.99–1.04)	0.188			1.07 (1.00–1.14)	0.047		
Congestive heart failure	1.48 (1.19–1.83)	<0.001	1.02 (0.78–1.33)	0.901	1.57 (0.95–2.58)	0.078	1.27 (0.67–2.42)	0.468
Hypertension	1.16 (1.00–1.35)	0.054			1.09 (0.73–1.65)	0.666		
Diabetes	1.04 (0.84–1.28)	0.712			0.85 (0.45–1.61)	0.611		
Stroke	1.05 (0.93–1.17)	0.445			1.08 (0.82–1.41)	0.579		
Vascular disease	1.02 (0.81–1.27)	0.898			1.13 (0.58–2.22)	0.715		
CHA_2_DS_2_-VASc score	1.07 (1.03–1.12)	0.002	0.99 (0.93–1.06)	0.840	1.07 (0.94–1.22)	0.299	0.97 (0.81–1.17)	0.775
LA diameter, mm	1.05 (1.04–1.06)	<0.001	1.04 (1.02–1.06)	<0.001	1.03 (0.99–1.06)	0.123	1.02 (0.98–1.07)	0.276
LAVI, ml/m^2^	1.03 (1.02–1.03)	<0.001			1.01 (0.99–1.02)	0.381		
LVEF, %	0.99 (0.98–1.00)	0.098			0.98 (0.96–1.01)	0.140		
E/Em	1.02 (1.00–1.03)	0.048	0.98 (0.96–1.01)	0.159	1.03 (0.98–1.08)	0.286	1.01 (0.95–1.08)	0.669
LA volume (by CT), ml	1.01 (1.01–1.01)	<0.001			1.00 (1.00–1.01)	0.095		
Pericardial fat volume (by CT), ml	1.00 (1.00–1.00)	0.839			1.00 (1.00–1.01)	0.276		
Prescence of extra PV foci	1.95 (1.59–2.39)	<0.001	1.91 (1.54–2.38)	<0.001	2.32 (1.52–3.56)	<0.001	2.68 (1.63–4.42)	<0.001

*Factors significant in the univariable analyses (P < 0.05) were entered into the multivariable analyses*.

*BMI, body mass index; CI, confidence interval; CT, computed tomography; HR, hazard ratio; E/Em; ratio of the peak mitral flow velocity of the early rapid filling to the early diastolic velocity of the mitral annulus; LA, left atrium; LAVI, left atrial volume index; LVEF, left ventricular ejection fracture; PV, pulmonary vein*.

## Discussion

### Main Findings

In this single-center, retrospective cohort study, we analyzed the isoproterenol-induced extra-PV triggers during *de novo* and repeat AF ablation procedures. Extra-PV triggers were more commonly found in the repeat ablation than in the *de novo* procedure. Older age and LA remodeling were independently associated with the presence of extra-PV triggers in the *de novo* ablation and being a woman, having diabetes, and higher parasympathetic nerve activity were related to the presence of extra-PV triggers in the repeat procedure. One-third of the extra-PV triggers newly found in the repeat ablation matched the empirical extra-PV ablation sites in the *de novo* procedure, and the existence of extra-PV triggers was independently associated with a higher AF recurrence after both the *de novo* and repeat ablation procedures.

### Potential Mechanisms of AF Recurrence After AFCA

Continuous long-term AF recurrence after AFCA is a current unmet need. Representative mechanisms of AF recurrence after ablation are PV reconnections, extra-PV triggers, and autonomic neural effects. We expect that the long-lasting PVI issue will be overcome with the development of an upgraded and effective catheter technology (15). However, the extra-PV trigger issue has not only an elusive mechanism but also has limited treatment methods. In this study, we found a significant relationship between an extra-PV trigger and atrial remodelings, such as age and LA volume. The study of Keita Watanabe et al. (16) showed that being a woman, a lower body mass index (BMI <23.8 kg/m^2^), absence of hypertension, and ventricular diastolic dysfunction were independent predictors of extra-PV foci in patients with paroxysmal AF. In this study, the existence of extra-PV triggers in repeat ablations was associated with women, diabetes, and cardiac parasympathetic activity as well as the previous empirical extra-PV ablation sites (32.6%). This suggested that the autonomic nervous activity and previous ablation lesions play some role in extra-PV triggers and the recurrence mechanism. In particular, myofibroblasts, ion current changes, and gap junctional remodeling accompanying matrix remodeling or local fibrosis processes lead to electrophysiological changes such as in the membrane potential, conduction velocity, and refractoriness (17, 18). This atrial substrate remodeling contributes to AF initiation and maintenance mechanisms by the perpetuation of triggers or micro re-entry. Pericardial fat volume was smaller in the patients with extra-PV triggers than those without in this study. Pericardial fat is associated with arrhythmogenicity and AF recurrence in previous studies (19, 20). However, the existence of extra-PV triggers seemed to be more closely related to being a woman rather than to pericardial fat volume or body-mass index in this study. According to the recent study regarding sex differences in the mapping of repeat ablation procedures, extra-PV triggers were more significantly frequent in women than in men. Still, pericardial fat volume was substantially smaller in women (21).

### Mapping and Ablation of Extra-PV Triggers

The prevalence of extra PV triggers in the *de novo* AFCA is variable and ranged from 3.2 to 62%, depending on the provocation protocols and punctuality of the mapping procedures (22–24). Unlike PVs, which were anatomically distinct structures, the mapping and ablation method for extra-PV triggers was elusive. A randomized-controlled trial failed to show an improvement in the rhythm outcome by additional empirical ablation for complex fractionated electrograms or rotors that were mapped during sustained AF (25). However, the study of Lee et al. reported that an extra-PV trigger ablation mapped after an isoproterenol provocation significantly lowered the AF recurrence through a randomized clinical trial (26). The work of Kim et al. also reported that the extra-PV ablation lowered the recurrence rate, but the rhythm outcome of the patients who had an extra-PV trigger was significantly worse than that of the patients without, even after an extra-PV trigger ablation (14).

There were several limitations to isoproterenol-provoked extra-PV trigger mapping and ablation. First, immediate trigger mapping was difficult, and the accuracy was decreased when the 3D map was shaken, following the electrical cardioversion due to patient movement. Second, the 3D map defined the exit site of triggers, but the actual foci might exist in the epicardial layer or deep inside the septum. Third, the isoproterenol provocation protocol has not been verified or standardized. We raised the target heart rate to 120 bpm and then induced AF considering the β-blocker effect, but it has not been proven that this dose is appropriate. In this study, there were fewer extra-PV triggers in patients with vascular disease, which might reflect the potential bias of a more careful isoproterenol dosing considering the coronary risk.

### Future Directions

The presence of an extra-PV trigger is an important factor that must be overcome in determining long-term prognosis after AFCA. However, it was not easy to eliminate the extra-PV triggers in 11% of the *de novo* and 29% of the repeat procedures or 23% of multiple and 7% of unmappable foci utilizing a contact electrode catheter. Entire chamber mapping such as with the ECGi panoramic map could be a breakthrough for extra-PV mapping but has a limitation of localizing the foci on the septum, which was the most common extra-PV foci site in the current and previous studies (27). Since the AF driver map using computational modeling was an entire chamber map, it can be used as a guide for extra-PV trigger ablation (28). AADs with an appropriate dose are an alternative option to suppress extra-PV triggers by controlling the ion currents. If the presence of an extra-PV trigger could be predicted before the AFCA procedure, it would be useful in selecting an appropriate ablation catheter (balloon or RF), determining the need for an isoproterenol provocation test, and evaluating the prognosis after the procedure.

### Limitations

This study had several limitations. First, it was a single-center, retrospective observational cohort study that might have involved selection bias. Further multi-center, prospective studies should be conducted. Second, there was no uniform strategy for the extra PV trigger ablation, and we performed an empirical extra PV ablation based on the discretion of the operators. Finally, we included patients with an isoproterenol provocation test and excluded those who did not undergo a provocation test. This might have resulted in selection bias.

## Conclusion

Extra-PV triggers were commonly found in AF patients with older age, in women, and patients with LA remodeling, high parasympathetic nervous activity, and previous empirical extra-PV ablations. The existence of extra-PV triggers was independently associated with a higher recurrence after both the *de novo* and repeat ablations.

## Data Availability Statement

The raw data supporting the conclusions of this article will be made available by the authors, without undue reservation.

## Ethics Statement

The studies involving human participants were reviewed and approved by the Institutional Review Board at Yonsei University Health System. The patients/participants provided their written informed consent to participate in this study.

## Author Contributions

H-NP contributed to the conception and design of the work and critical revision of the manuscript. DK and TH contributed to the conception and design of the work, interpretation of data, and drafting of the manuscript. MK, HY, T-HK, and J-SU contributed to the acquisition and analysis of data. BJ and M-HL contributed to the conception and design of the work and revision of the manuscript. All authors contributed to the article and approved the submitted version.

## Funding

This study was supported by grants (HI19C0114) and (H21C0011) from the Ministry of Health and Welfare and a grant (NRF-2020R1A2B01001695) from the Basic Science Research Program run by the National Research Foundation of Korea (NRF), which was funded by the Ministry of Science, ICT, and Future Planning (MSIP).

## Conflict of Interest

The authors declare that the research was conducted in the absence of any commercial or financial relationships that could be construed as a potential conflict of interest.

## Publisher's Note

All claims expressed in this article are solely those of the authors and do not necessarily represent those of their affiliated organizations, or those of the publisher, the editors and the reviewers. Any product that may be evaluated in this article, or claim that may be made by its manufacturer, is not guaranteed or endorsed by the publisher.
