# Supplementary material for: Extra-Pulmonary Vein Triggers at de novo and the Repeat Atrial Fibrillation Catheter Ablation
Source: Front Cardiovasc Med. 2021 Nov 4;8:759967. doi: 10.3389/fcvm.2021.759967 (PMC8600078; doi:10.3389/fcvm.2021.759967)
Supplement: Supplementary file 1 [file Data_Sheet_1.docx]

**Supplementary Table 1.** Complications after the de novo catheter ablation of atrial fibrillation.

|  | **All patients**  **(n=2119)** | **Extra-PV triggers** | | | **Use of CF-sensing catheter** | | |
| --- | --- | --- | --- | --- | --- | --- | --- |
| **Complications** |  | **(-)**  **(n=1871)** | **(+)**  **(n=247)** | **P value** | **Pre CF-sensing era**  **(n=1088)** | **CF-sensing era**  **(n=1030)** | **P value** |
| Overall complications | 75 (3.5) | 62 (3.3) | 13 (5.3) | 0.169 | 44 (4.0) | 31 (3.1) | 0.242 |
| Major complication* | 34 (1.6) | 28 (1.5) | 6 (2.4) | 0.408 | 18 (1.7) | 16 (1.6) | 0.990 |
| Atrioesophageal fistula | 1 (0.0) | 1 (0.1) | 0 (0.0) |  | 1 (0.0) | 0 (0.0) |  |
| Vascular access complication | 16 (0.8) | 15 (0.8) | 1 (0.4) |  | 9 (0.8) | 7 (0.7) |  |
| Cardiac tamponade/hemopericardium | 19 (0.9) | 16 (0.9) | 3 (1.2) |  | 11 (1.0) | 8 (0.8) |  |
| Pulmonary vein stenosis | 5 (0.2) | 4 (0.2) | 1 (0.4) |  | 1 (0.0) | 4 (0.4) |  |
| Phrenic nerve paralysis | 7 (0.3) | 5 (0.3) | 2 (0.8) |  | 2 (0.2) | 5 (0.5) |  |
| Stroke/transient ischemic attack | 2 (0.1) | 2 (0.1) | 0 (0.0) |  | 2 (0.2) | 0 (0.0) |  |
| Complete atrioventricular block | 2 (0.1) | 2 (0.1) | 0 (0.0) |  | 2 (0.2) | 0 (0.0) |  |
| Pericarditis | 8 (0.4) | 5 (0.3) | 3 (1.2) |  | 6 (0.6) | 2 (0.2) |  |
| Others† | 25 (1.2) | 20 (1.1) | 5 (2.0) |  | 16 (1.5) | 9 (0.9) |  |

Values are presented as number (%). CF, contact force; PV, pulmonary vein.

*Complications that resulted in permanent injury or death, required intervention for treatment, or a prolonged or required hospitalization for more than 48 hours.

†Includes pleural effusion, shock due to unknown etiology, sudden cardiac arrest, and sinus node dysfunction.

**Supplementary Table 2.** Complications after the repeat ablation of atrial fibrillation.

|  | **All patients**  **(n=227)** | **Extra-PV triggers** | | | **Use of CF-sensing catheter** | | |
| --- | --- | --- | --- | --- | --- | --- | --- |
| **Complications** |  | **(-)**  **(n=162)** | **(+)**  **(n=65)** | **P value** | **Pre CF-sensing era**  **(n=73)** | **CF-sensing era**  **(n=154)** | **P value** |
| Overall complications | 10 (4.4) | 4 (2.5) | 6 (9.2) | 0.059 | 4 (5.5) | 6 (3.9) | 0.844 |
| Major complication* | 5 (2.2) | 2 (1.2) | 3 (4.6) | 0.285 | 1 (1.4) | 4 (2.6) | 0.917 |
| Atrioesophageal fistula | 0 (0.0) | 0 (0.0) | 0 (0.0) |  | 0 (0.0) | 0 (0.0) |  |
| Vascular access complication | 0 (0.0) | 0 (0.0) | 0 (0.0) |  | 0 (0.0) | 0 (0.0) |  |
| Cardiac tamponade/hemopericardium | 3 (1.3) | 2 (1.2) | 1 (1.5) |  | 2 (2.8) | 1 (0.6) |  |
| Pulmonary vein stenosis | 1 (0.4) | 1 (0.6) | 0 (0.0) |  | 0 (0.0) | 1 (0.6) |  |
| Phrenic nerve paralysis | 1 (0.4) | 1 (0.6) | 0 (0.0) |  | 0 (0.0) | 1 (0.6) |  |
| Stroke/transient ischemic attack | 0 (0.0) | 0 (0.0) | 0 (0.0) |  | 0 (0.0) | 0 (0.0) |  |
| Complete atrioventricular block | 0 (0.0) | 0 (0.0) | 0 (0.0) |  | 0 (0.0) | 0 (0.0) |  |
| Pericarditis | 0 (0.0) | 0 (0.0) | 0 (0.0) |  | 0 (0.0) | 0 (0.0) |  |
| Others† | 5 (2.2) | 0 (0.0) | 5 (7.7) |  | 2 (2.8) | 3 (1.9) |  |

Values are presented as number (%). CF, contact force; PV, pulmonary vein.

*Complications that resulted in permanent injury or death, required intervention for treatment, or a prolonged or required hospitalization for more than 48 hours.

†Includes sinus node dysfunction.

**Supplementary Table 3.** Locations of the extra-PV triggers

| **Focused on Each Patient*** | **Overall**  **(n=290)** | ***De novo* AFCA**  **(n=231)** | **Repeat AFCA**  **(n=59)** | **p-value** |
| --- | --- | --- | --- | --- |
| **Mean number of mappable extra-PV foci per patient** | 1.27 ± 0.54 | 1.22 ± 0.46 | 1.49 ± 0.73 | <0.001 |
| Single focus, n (%) | 223 (76.9%) | 186 (80.5%) | 37 (62.7%) | <0.001 |
| Double foci, n (%) | 56 (19.3%) | 40 (17.3%) | 16 (27.1%) |  |
| Triple foci, n (%) | 10 (3.4%) | 5 (2.2%) | 5 (8.5%) |  |
| ≥ four foci, n (%) | 1 (0.3%) | 0 (0%) | 1 (1.7%) |  |
| **Main extra-PV trigger site per patient** | | |  | 0.092 |
| Septum | 62 (19.9%) | 50 (20.2%) | 12 (18.5%) |  |
| Coronary sinus | 46 (14.7%) | 35 (14.2%) | 11 (16.9%) |  |
| SVC | 35 (11.2%) | 32 (13%) | 3 (4.6%) |  |
| Crista terminalis | 21 (6.7%) | 18 (7.3%) | 3(4.6%) |  |
| LA posterior wall | 15 (4.8%) | 12 (4.9%) | 3 (4.6%) |  |
| LAA and LOM area | 13 (4.2%) | 12 (4.9%) | 1 (1.5%) |  |
| Bachmann’s bundle area | 10 (3.2%) | 8 (3.2%) | 2 (3.1%) |  |
| RA sinus venosa | 5 (1.6%) | 3 (1.2%) | 2 (3.1%) |  |
| Other sites | 16 (5.1%) | 16 (6.5%) | 0 (0%) |  |
| Multifocal triggers | 67 (21.5%) | 45 (18.2%) | 22 (33.8%) |  |
| **Focused on extra-PV triggers***  **(Number of extra PV foci)** | **Overall**  **(n=369)** | ***De novo* AFCA**  **(n=281)** | **Repeat AFCA**  **(n=88)** |  |
| **Extra-PV trigger site** |  |  |  | 0.185 |
| Septum | 100 (27.1%) | 73 (26.0%) | 27 (30.7%) |  |
| Coronary sinus | 77 (20.9%) | 56 (19.9%) | 21 (23.9%) |  |
| SVC | 60 (16.3%) | 49 (17.4%) | 11 (12.5%) |  |
| Crista terminalis | 30 (10.3%) | 24 (8.5%) | 6 (6.8%) |  |
| LA posterior wall | 23 (6.2%) | 17 (6.0%) | 6 (6.8%) |  |
| LAA and LOM area | 24 (6.5%) | 19 (6.8%) | 5 (5.7%) |  |
| Bachmann’s bundle area | 20 (5.4%) | 13 (4.6%) | 7 (8.0%) |  |
| RA sinus venosa | 7 (1.9%) | 5 (1.8%) | 2 (2.3%) |  |
| Other sites | 28 (7.6%) | 25 (8.9%) | 3 (3.4%) |  |

*Patients with unmappable extra-PV foci were excluded in the analysis.

AFCA, atrial fibrillation catheter ablation; LA, left atrium; LAA, left atrial appendage; LOM, ligament of Marshall; PV, pulmonary vein; RA, right atrium; SVC, superior vena cava.

**Supplementary Figure 1**. Receiver operating characteristic curves of left atrial diameter for clinical recurrence after *de novo* ablation for AF showing the capacities and optimal cut-off values.


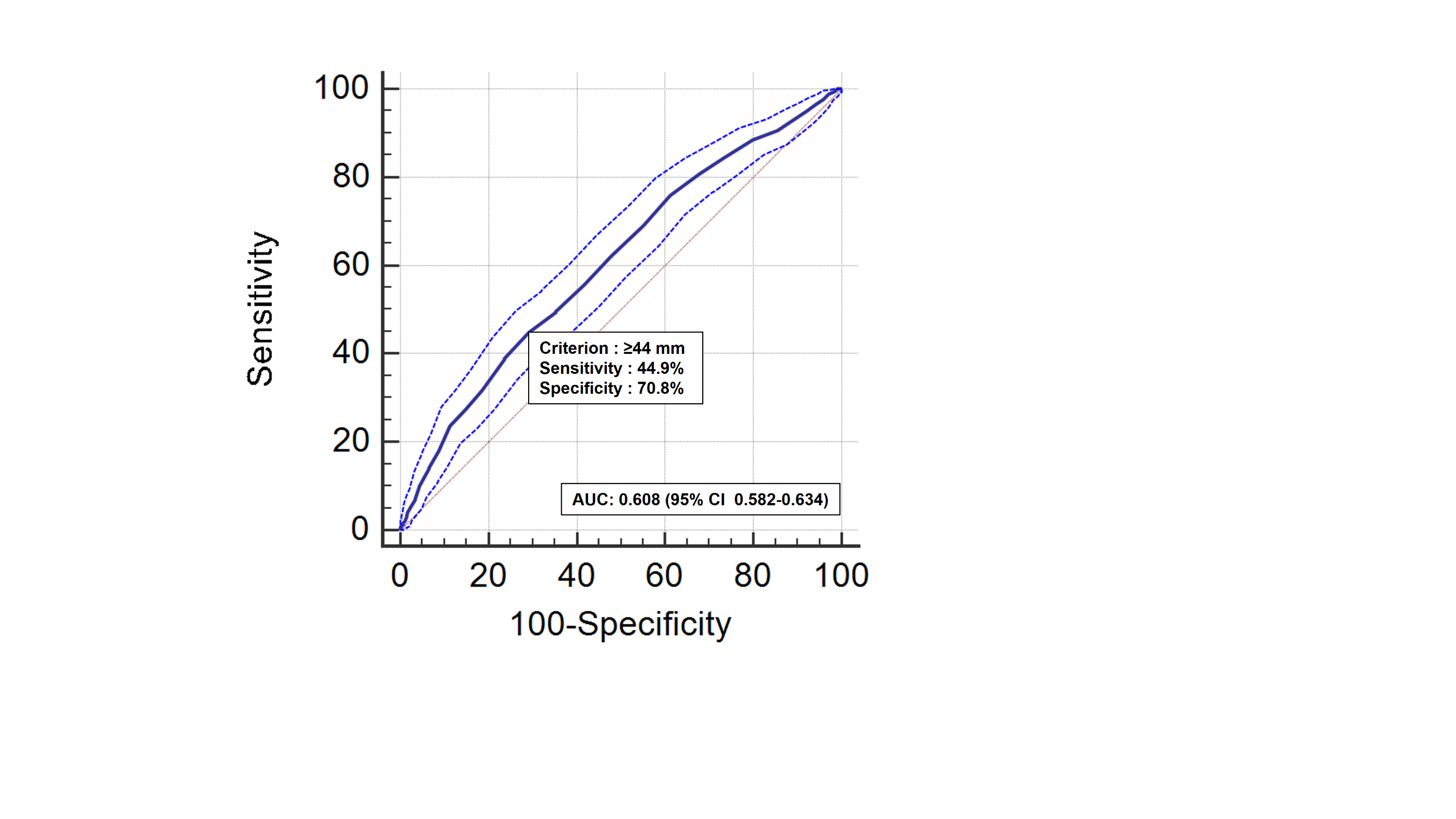


AUC, area under curve, CI, confidence interval.
